# Language Development for the New Generation of Children with Hearing Impairment

**DOI:** 10.3390/jcm10112350

**Published:** 2021-05-27

**Authors:** Lone Percy-Smith, Signe Wischmann, Jane Lignel Josvassen, Christina Schiøth, Per Cayé-Thomasen

**Affiliations:** 1 Copenhagen Hearing and Balance Center, Ear, Nose and Throat (ENT) and Audiology Clinic, Rigshospitalet, Copenhagen University Hospital, DK-2100 Copenhagen, Denmark; per.caye-thomasen.01@regionh.dk; 2 Patient Organisation, Decibel, DK-2100 Copenhagen, Denmark; signe@decibel.dk (S.W.); jane@decibel.dk (J.L.J.); christina@decibel.dk (C.S.)

**Keywords:** pediatric hearing impairment, long term language outcomes, social well-being, early intervention, auditory verbal therapy

## Abstract

A new generation of children with hearing impairment (HI) has emerged due to the introduction of universal neonatal hearing screening, medical–surgical/technical and educational advances. Aim: Investigation of long-term development of vocabulary and social well-being of children with HI, including children with HI and additional disability. Method and Material: The project design was prospective, longitudinal, and comparative. Level of receptive vocabulary was compared to children with normal hearing, type of hearing technology, gender, additional disability, diagnosis of HI, level of social well-being, and start age for use of hearing technology. A total of 231 children participated. Intervention included early start of hearing technology and three years of auditory–verbal therapy (AVT) at the preschool level, followed by 3 years of AV guidance at the school level. Results: Children with HI scored within the norm for receptive vocabulary but were outperformed by the control group. Children with HI and a diagnosed additional disability scored lower than children without additional disability, in terms of parental assessments of social well-being. Children with additional disabilities showed positive progression in terms of receptive vocabulary development. Conclusions: New generations with HI possess the potential to succeed academically in accordance with individual abilities and become active participants in the working market.

## 1. Introduction

The introduction of universal neonatal hearing screening (UNHS), digital hearing aids (HA), and cochlear implants (CI) for pediatric populations with hearing impairment (HI) has improved the life conditions for children with all degrees of HI. Former generations of children with HI are described to not have succeeded academically, and 51% of adults with profound HI are not active participants in the working market [1]. However, for the new generation of children with HI, it has been documented that early intervention with hearing technology and enrolment in family-centered, auditory–verbal intervention, allow children to close the language gap and develop age-equivalent language before entering school [2]. The efficacy of AV intervention has been documented in terms of language development, and children with HI are now able to perform at levels equivalent to their normal hearing peers [3]. However, clinical experience has also shown that various degrees of sensory deprivation in the prenatal period can have a profound and permanent effect on the development of the entire central auditory system [4]. Furthermore, the HI may influence how children with HI acquire language, which differ from normal hearing peers [5]. Children who acquire a HI, for instance due to meningitis, experience drastic change due to sensory loss, at later stages of life. Therefore, it is important to keep monitoring pediatric populations with HI in terms of all aspects of audition and language development [6]. A recent study underlined this notion as it was found that early implanted children progressed really well in the first years after implantation, but after four years, a gap between children with CI as compared to children with NH started growing in terms of development of receptive vocabulary [7].

Congenital HI is one of the most frequent functional disabilities in our society. It is estimated that severe-to-profound HI affects one child in every 2000 every year [8]. In order to fully understand pediatric audiology, it is, furthermore, important to recognize that 95% of children with HI are born into families with normal hearing (NH). Therefore, parents have a strong incentive to pursue a listening and spoken language intervention [9], which can only be done by use of hearing technology, e.g., HAs, CIs, bone-anchored hearing aids (Bahs), and auditory brainstem implant (ABI). 

HI often adversely affects speech and language, especially in children at ages before and during language acquisition, because their auditory pathways and language are still developing [10]. In young children, HI may cause delayed or decreased speech and language development and compromised communicative function. When children develop language, they need to have access to the whole frequency range (speech range) to properly develop speech intelligibility and speech understanding, to develop reading and writing skills, and learn other languages [11]. Even though HI is amplified with state-of-the-art hearing technology, these children do not have normal hearing. Distance to the sound signal and noise in the child’s surroundings are “enemies” that will lead to less overhearing. Overhearing is the incidental listening to conversations of others and thereby learning the rules of language, through repeated exposures in different contexts. Overhearing accounts for up to 90% of daily language learning [12]. Lack of overhearing will lead to reduction in development of age-appropriate vocabulary, grammatical skills, knowledge of the world, pragmatic skills, and social interaction. Hence, putting the children at risk of microsocial exclusion. Therefore, it is pertinent to offer primary state-of-the-art hearing technology, guidance to parents, and other professionals around the child, and to provide additional technical solutions to diminish the impact of the HI.

Furthermore, HI has an impact on the aspects of socio-emotional development, e.g., behavior, energy, stress, self-esteem, and anxiety, consequently leading to problems with maintaining a normal social life. This is alarming, because social interactions and friendships are associated with psychosocial well-being as well as having close, positive peer relationships, which is associated with increased self-esteem, regulation of emotion, better adjustment to school, and a positive attitude to school. Hence, HI can have a substantial negative and lifelong impact on Quality of Life (QoL), including social well-being. Therefore, longitudinal studies of language development of pediatric populations with HI must always include investigation of the levels of social well-being [13]. Furthermore, there are documented significant associations between level of language and level of QoL [14,15,16]. 

The documented positive effect of early medical-surgical and technical intervention, combined with specific auditory-verbal training at pre-school level [11,12], must be followed by studies monitoring language level at later developmental stages. This importance is underlined by the fact that students with profound HI have traditionally attended special institutions for the deaf. However, due to the improvements in the field, most children with HI are today placed in mainstream schools. This shift in educational placement is highly cost-beneficial to society, and furthermore, is associated with better overall language outcomes for children with HI. Language outcomes refer to all aspects of language, e.g., vocabulary, grammatical and lexical understanding, pragmatics, and phonology [7,14]. Over the past decades, it has been documented that with early technical and educational intervention, children with HI are able to acquire language within the normal range [17,18,19]. Moreover, it is important to investigate how children get on in mainstream educational settings, as challenges have been documented [20].

### Aim

The primary aim of the study was to investigate development of receptive vocabulary, over a six-year period, for the new generation of children with HI at the preschool level and into the first three years of school. A secondary aim was to investigate level of social well-being both at preschool and school level. Furthermore, the study investigated possible differences and similarities between children using different hearing technology, (e.g., HA/Bahs, CI) and analyzed possible associations between the level of vocabulary and social well-being. The study involved children with HI and additional disability, and it was an aim to study these children separately, in terms of level of receptive vocabulary and level of social well-being associated with type of HI and other disability.

## 2. Materials and Methods

The overall project design was of a methodological character, and thereby, excluded issues of an experimental nature, and therefore, ethical approval was not required. The project was conducted in strict accordance with the “Danish Code of Conduct for Research Integrity” from the Ministry of Higher Education and Science. All testing of children was only carried out when there was a signed approval from the child’s family or legal caregiver. The project design was prospective, longitudinal, and comparative, and was conducted from September 2013 to December 2020. Level of receptive vocabulary was compared to type of hearing technology, gender, additional disability, diagnosis of HI, level of social well-being, and age at start of use of hearing technology.

### 2.1. Intervention

The rehabilitation of the children with HI involved both technical intervention (HA, CI, Bahs) and Auditory–Verbal (AV) intervention. The AV intervention was an educational intervention that specifically targeted children with HI, regardless of the degree of HI and the type of hearing device. AV intervention underlines the importance of parents and professionals working closely in partnership and makes use of specific techniques and strategies to develop and grow the child’s auditory cortex toward the preferred listening and spoken-language outcomes. AV practice is defined as a family-centered approach and an applied science, with its objectively measured goals [21]. At the preschool level, children and families received AV intervention every other week, monthly or every other month, depending on the child’s and the family’s individual progress. AV intervention was carried out by speech and language pathologists, who were either certified AV practitioners or who had completed the 3-year AV education provided by the AG Bell Academy for Listening and Spoken Language [22]. At the school level children, families and teachers received AV guidance, which included, annual testing in terms of level of audition/listening, speech, language, pragmatics, and social well-being; annual information meetings for parents and teachers about school issues (such as how to use hearing assistive devices and how to implement auditory–verbal strategies and techniques in the classroom); and bi-annual visits to each child’s school with systematic observations in classrooms, followed by supervision of the teachers.

### 2.2. Applied Tests

The present study investigated the development of the children’s receptive vocabulary through use of the Peabody Picture Vocabulary Test, 4th edition (PPVT-4) [23]. The PPVT-4 is a widely used norm-referenced test of receptive vocabulary. PPVT-4 is standardized on data from approximately 3500 subjects from the US. The sample matches the U.S. Census for gender, race/ethnicity, region, socioeconomic status (SES), and clinical diagnosis or special education placement. The Danish version of PPVT-4 was translated over a period of 1.5 years, by two speech and language pathologists. The Danish translations were translated back to English by two bilingual (English/Danish) persons. The pictures shown to the children were not altered and as there was no Danish standardization of the PPVT-4; the Danish children with HI were scored according to scores from American children. To make up for the lack of a Danish norm, a large control study involving 173 children with normal hearing (NH) was carried out. The control group was matched according to gender, age, and demography. Children in the control group were tested once during the project, while children with HI were tested each year over the six-year project period. During a test, the respondents were required to point to one of four pictures that represent the word produced by the tester. Standard scores are defined as +/− 1 standard deviation. 

Parents reported on their child’s social well-being twice during the project. The first time-point was after three years of AVT and the second time-point was after six years of AV guidance. This is referred to as year 3 and year 6 throughout the paper. Parents assessed their child’s level of social well-being according to a scale used on pediatric populations with NH [24]. Social well-being was defined in terms of self-esteem parameters. The assessment consisted of a seven-point rating scale to determine the degree of the child’s personal–social adjustment, by assessing whether the child was dependent vs. independent, passive vs. active, lonely vs. social, worried vs. not worried, sad vs. happy, and insecure vs. confident. In accordance with the defined score by the National Institute of Public Health, a score below 36 was defined as a low level of social well-being and a score above 36 was defined as a high level of social well-being. Maximum score = 42 and minimum score = 7. 

Information on type of hearing technology, gender, additional disability, diagnosis of HI, and age at start of use of hearing technology was retrieved from parental questionnaires and the child’s medical record.

### 2.3. Material

A total of 231 children participated in the project (*n* = 58 HI, *n* = 173 NH). Approximate number of parents and teachers of children with HI was 250 over the six-year project period. Children were born from 2008 to 2013 and were between 0–4 years of age at the start of the project in September 2013. All children with CI were implanted at one of the two pediatric CI centers in Denmark and all children with HA/Bahs were enrolled at the two major audiological clinics in Denmark, i.e., university hospitals at Rigshospitalet and Aarhus.

Table 1 summarizes the background variables for children with HI and NH. Children with NH (*n* = 173) are described in terms of gender. Out of a total of 58 children, 42 children participated in both the preschool project and the school project, and 25 (43%) children completed the PPVT-4 every single year. Some children were too young to perform the PPVT-4 every year at the preschool level, and some children were not testable every year, which explained the different sample sizes. The children came from Nordic countries but with a vast majority from Denmark (*n* = 55), Faroe Islands (*n* = 1), Sweden (*n* = 1), and Norway (*n* = 1). The children from Faroe Islands, Sweden, and Norway visited for AV sessions every other month and some sessions were conducted via telepractice. In the school project, children and family visited every 3 months and the AV practitioner visited the schools once every year. Teachers and parents participated virtually at the annual meetings.

In the literature, children with HI and additional disability are described in various ways. Cupples et al. (2013) highlight the effect of different types of additional disabilities on language development, in children with HI [25] and Zaidman-Zait et al. (2017) introduced the terms CI-TD and CI-DD in a study referring to two groups of children with CI; one group without additional disabilities and one group with additional disabilities [26]. These approaches introduced a more nuanced way of describing children with additional disabilities. However, the number of children with additional disabilities in our study was not large enough to make such groupings, and hence, we compared children with HI with or without additional disability. The types of disabilities included—visual disabilities, speech-output disabilities, attention deficit/hyperactivity disorder (ADHD) and medical disabilities, autism spectrum disorder, cerebral palsy, developmental delay, CHARGE syndrome, Noonan syndrome, microcephaly, and Asphyxia. PPVT-4 results from children with additional disability were included in the analysis of the long-term vocabulary development but data for this group was also analyzed separately, both in terms of language development and social well-being. 

The preschool project was funded by the Ministry of Social Affairs and the school project was funded by the Innovation Fund Denmark and the William Demant Foundation. It was free of charge for participants and schools, but time off from work and transportation were paid by the families and schools. Thirty-six local municipalities were represented, and 42 schools participated with one or two teachers. At the preschool level, four families declined for various reasons; two families chose another AV program and never participated in the project, one family was guided by the AV practitioner to pursue a sign language intervention, and one family dropped out after one year due to personal reasons. A total of 58 families started in the preschool project and 55 (95%) families completed all 3 years of AV intervention. Inclusion criteria for the school project was that the child and family had 3 years of AV intervention prior to starting school and were fulltime users of bilateral hearing technology. Countrywide, 54 children with HI and their families fulfilled these criteria and 47 accepted the invitation to participate (participation rate = 87%). No common denominator in terms of gender, age, and technology was found for the seven children/families who declined participation. Halfway through the school project, one family with a child with additional disabilities chose to pursue a sign language intervention and hence was guided by an AV practitioner to resign from the project. Completion of the school project was 98%. 

### 2.4. Statistical Analysis 

Descriptive statistics were summarized as frequencies and percentages. The group of children with HI, including children with HI and additional disability, was compared to a group of children with NH, by comparing the mean values of the PPVT-4 standard scores using one-sided independent samples *t*-test. To test for correlation between PPVT-4 standard scores, social well-being, hearing device, and additional disability, the Pearson’s chi-square test was applied because of its robustness with respect to the distribution of data. In the correlation table, data are presented as grouped, but analysis was made on longitudinal data. The standard scores, SD, were grouped as follows: <85; 85–100; >100. *p*-values below 0.05 were considered to be statistically significant. Analyses were performed using the SAS software 9.4 (SAS Institute Inc., Cary, NC, USA).

## 3. Results

Table 1 shows the results in terms of age at diagnosis and age at time of intervention with hearing technology, for all children (*n* = 58). Children with a diagnosed additional disability represented a total of 34% (*n* = 20). The children scored high on the level of social well-being, e.g., 68%, both after the first 3 years of the project and after 6 years. In comparison, 58% of children with NH scored at a high level [24]. The age of intervention with hearing technology was similar for the children with CI or HA/Bahs, e.g., 13 months. 

**Figure 1:**
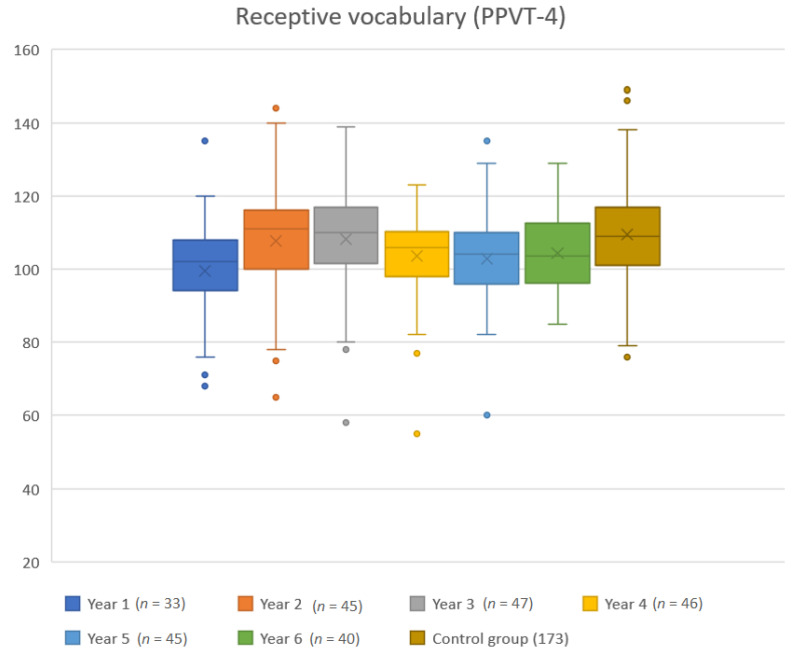
Annual standard scores of children with HI as compared to the control group of children with NH. Dots are outliers. Mean standard score according to the American norm is 100 and standard deviation is 15.

Figure 1 summarizes the standard scores of the PPVT-4 in box plots. Median scores are presented as a horizontal line in each box, mean scores as X, and outliers are presented as dots. Overall, the standard scores are markedly high. When compared to the control group, children with HI were outperformed at year 1 (*p* = 0.0004). For year 2 and 3, there were no statistically significant differences between the groups. However, at year 4, 5, and 6, children with HI were outperformed by children with NH, at statistically significant levels, *p* = 0.0040, *p* = 00013, and *p* = 0.0014, respectively.

Cross-tabulations are summarized in Table 2 showing the analyses of possible differences and similarities between children with CI versus children with HA/Bahs, in terms of standard scores from PPVT-4, as compared to the level of social well-being at year 3 and year 6. There were no statistically significant differences between a standard score <85 and a low level of social well-being at year 3 or 6. No children with HI scored at the lowest level <85 in the standard scores of the PPVT-4 and the low-levels of social well-being at year 6. 

The analysis of type of device, HA/Bahs versus CI, compared to standard scores on the PPVT-4, revealed no statistically significant difference, either at year 3 or year 6.

**Figure 2:**
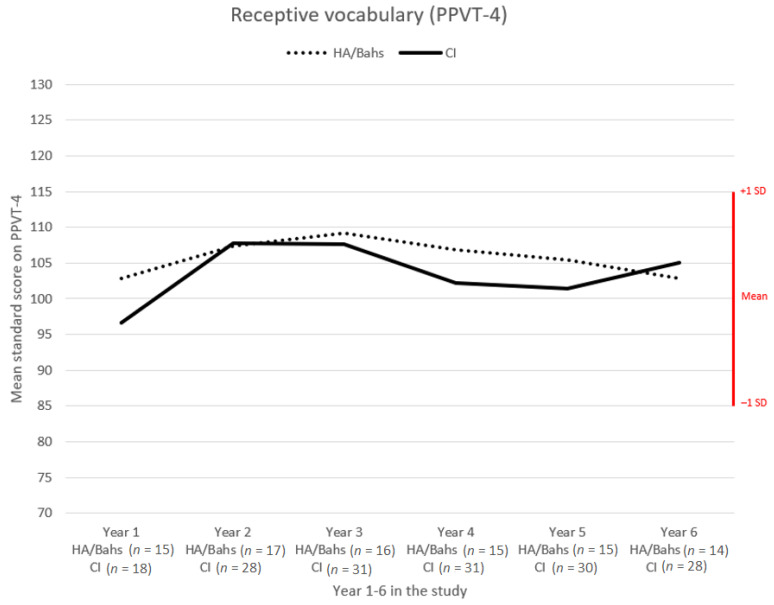
Comparison of mean standard scores on PPVT-4 of children with different devices over the 6-year project period.

Even though the statistical analysis did not find any difference between level of vocabulary and type of device, it is interesting to further study the scores of children with different devices. Figure 2 provides an overview of the scores for children with HA/Bahs versus children with CI. Children with HA/Bahs scored higher every year except for year 6. The slopes of the curves were similar but with a noticeable dip at year 4, which is the age of school start.

**Figure 3:**
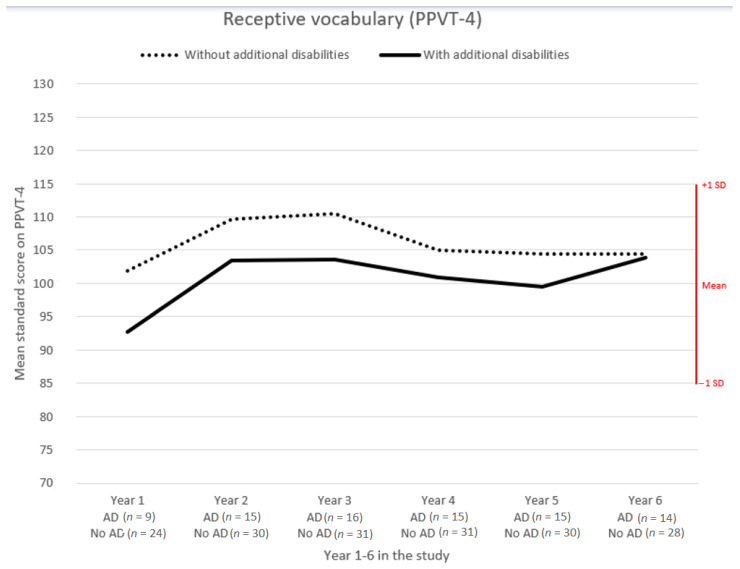
Comparison of mean standard scores on PPVT-4 of children with and without additional disabilities (AD), over the 6-year project period.

Results from the PPVT-4 were analyzed separately for children with HI and additional disability and were compared with children without a diagnosed additional disability. Figure 3 illustrates the development for the two groups, and it is noteworthy that children with additional disability showed a slower but positive development over the six-year period. 

The analyses in terms of parental assessments of level of social well-being was resumed in two categories and summarized into a low vs. a high level of social well-being. Table 3 summarizes the spread of data. The statistical analysis found a significant difference between children with and children without additional disability (*p* = 0.0039) at year 6. Children with HI and additional disability were assessed to have a lower level of social well-being.

## 4. Discussion

The design of the study did not allow determination of causality among variables, but it explored how children acquire receptive vocabulary when type of intervention, hearing technology and AV, were kept constant in a longitudinal study. For ethical reasons, it was not possible to randomize or match children with HI who were randomly placed in an AV intervention group versus a non-intervention group. Furthermore, it is questionable whether parents participate in such studies. In Denmark, with highly educated parents, such studies would probably have been difficult to conduct. 

The Early Hearing Detection and Intervention (EHDI) guidelines recommend hearing screening by one month, diagnosis of hearing loss by 3 months, and intervention by 6 months. Intervention is defined as both technical, e.g., by fitting of HAs and educational, e.g., providing guidance to parents in early communication with a baby with HI [27]. The median age of intervention with hearing technology was 13 months for children with HA/Bahs, which is seven months later than the EHDI guidelines. It is relevant to discuss this result, considering the sample size, and it is questionable whether the 19 children with HA/Bahs were representative of children with HA/Bahs in Denmark. This result calls for larger studies of the effect of UNHS. All children with CI in Denmark must have a trial period with HA before implantation, e.g., children with CI start intervention with hearing technology earlier than children with HA/Bahs. A median implantation age of 13 months was a positive result. Therefore, it can be argued that in our study, it seemed that children with congenital severe-to-profound HI were better off in terms of start age of intervention than children with slight to moderately severe HI. It may be argued that severe to profound congenital HI regardless of etiology is a more straightforward process to diagnose, and therefore, the intervention also starts earlier. Moderate HI due to Pendred’s syndrome presents with challenges of fluctuations that can be hard to identify in young children, and therefore, can be challenging to fit with hearing technology. Nevertheless, the identified difference between the two groups makes future studies highly warranted. Despite a fairly late start age for children with HA, the children tend to perform higher than children with CI in terms of vocabulary development, but not to a statistically significant level. Therefore, it is important to keep monitoring the language development of the new generation of children with HI, also seen in the light of long-term language outcome studies that indicate that the effect of early implantation age diminishes with time, particularly for higher-order language skills, such as reading [28]. 

The prevalence of children with HI and additional disability varies in the literature, but several studies report that a prevalence of approximately 30–40% of children with sensorineural HI > 40 dB have additional disabilities [8,29]. Inspired by the studies of Cupples et al. [25] and Zaidman-Zait et al. [26], our study sought to nuance the perception of children with HI and additional disability, but due to the small sample size, we chose not to pursue any of these groupings. However, from an ethical perspective, it is positive to nuance perception, and such classifications may support children with HI and additional disability, to pursue development of audition and language aligned with each child’s individual ability and potential. In our study, a progressive and positive development in terms of language development was documented for children with HI and additional disability. This indicates that with on-going technical and educational intervention, some of these children can acquire receptive vocabulary within the norm. It can be argued that our study design implied a limitation in terms of alignment with the existing literature on the prevalence of children with HI and additional disabilities, as it was unknown to what extent the children may have a non-recorded additional disability that will surface with time. An additional disability may affect language development even at a time, when not yet diagnosed. This could be the case for a congenital deaf child with CI and additional disability, e.g., dyslexia. Nevertheless, it seems highly warranted for future studies of children with HI and additional disability, to investigate both prevalence and the children’s ability to develop listening and spoken language in a long-term perspective. Our study found a statistically significant difference between children with HI with additional disability and without additional disability. Therefore, studies of children with HI and additional disability should not be restricted to speech production alone. It is relevant to investigate aspects of quality of life, as spoken language development is not always the goal for these children, but rather it may be an overall goal to increase quality of life by enabling the child’s access to sound from hearing technology. It is stressed that an observational questionnaire is often the only possible way of monitoring outcome for these children, as other existing tests involve tasks too complex [30]. Such questionnaires, together with a more nuanced perception, will strengthen developmental opportunities for children with HI and additional disabilities.

The very high standard scores of the PPVT-4 for all children were noticeable. This may reflect the fact that the standard scores were based on American norms and not on Danish norms. This is a problem for small countries, because standardisation is time-consuming and expensive to carry out and publishers might not want to invest in small countries with a limited market. Another challenge in translating and culturally modifying speech and language tests is balancing the level of difficulty between two languages. Therefore, it is of high importance to conduct a control group of children with NH as we have done in our study. Without knowledge from a direct comparison control group we could have concluded that children with HI in AV intervention outperform the norms. Instead, we can now show that the difference between children with HI and children with NH was significantly different at year 1, 4, 5, and 6 time of testing. At year 2 and 3, there was no significant difference between the HI group and the control group, and without a longitudinal study, it could have been concluded that children catch up and do not need AV guidance at the school level. Such conclusion would not have supported children with HI. Our results are aligned with results from a recent study [7] documenting that, over time, a gap in language performance seems to grow when children with HI are compared to normal hearing peers. This underlines the importance of carrying out longitudinal studies of children with all types and degrees of HI. This would provide updated documentation of a population, which due to technical and educational progress, has undergone drastic changes, and therefore faces different challenges in school. The slight dip in performance of children with HA/Bahs at year 6 would be interesting to study further, in order to investigate possible associations between sample size, limitation of both the technical and the AV intervention, and the limitation of a translated speech and language test. 

The participants of our study represent tomorrow’s generations of children with HI. They have been identified through UNHS, technical intervention with CI/HA/Bahs was (fairly) early, and they have received early educational intervention with Auditory–Verbal Therapy at the preschool level, followed by AV guidance into the first three years of school. As opposed to a recent study of QoL and language outcomes [14], our study investigated a cohort where the educational intervention was a constant parameter and we found that our cohort with HI performed high on language and social well-being parameters. Therefore, we stress that future studies of cohorts with HI should state the kind of educational approach, as well as parameters of age at implant and type of hearing technology. Furthermore, this is underlined by studies that throughout the years have documented the impact of type of intervention on all aspects of outcomes [6,9,31,32]. There is a need to keep investigating the existing cohorts of children with HI aged 9–12 years (tween age), in order to describe updated and relevant academic and social levels of tomorrows’ generations of individuals with HI. Such knowledge will enable the children to reach their full potential. Descriptions of children with HI need to be re-written and directly compared to populations with NH, and for instance, investigate test results from the National Curriculum, which has never been carried out in the Nordic countries.

Future studies of the new generation of children with HI should involve the children themselves. One way of further investigating the new generation with HI could be by including the children and adolescents with HI themselves, both in terms of studies of QoL/social well-being and in developing innovative hearing solutions and educational materials. Future studies should have a participatory design, which would allow for co-creation of various developments of technology and educational applications and self-reported questionnaires, securing direct inputs from the users themselves. Future data should be based on patient reported outcomes (PROs), which will ensure technical and educational developments that are aligned with today’s generation of children and adolescents with HI. Furthermore, this will result in better self-advocacy and meet the real needs of the new generation of children with HI.

## 5. Conclusions

Due to technical and educational advances, a new generation of children with HI has emerged. Our long-term study showed that with early technical and educational intervention, children with HI have the potential of developing receptive vocabulary within the normal range. However, when compared to a control group of children with NH, children with HI were significantly outperformed, and therefore, it must be underlined that ongoing monitoring of children and adolescents with HI are warranted. However, the Nordic children were scored according to an American norm and future studies should strive to incorporate norm data from Nordic children. Furthermore, a vast majority of children with HI, score high on parameters of social well-being, both at the preschool level and the school level. Our study suggests a classification of children with HI and additional disability to be more nuanced, in order to support each child’s individual potential to be exploited optimally.

Descriptions and perceptions of children with HI need to be re-written, in order to meet the true needs of future generations of children and adolescents with HI.

## Figures and Tables

**Table 1:** Background variables for children with HI and NH.

Background Variables	Children with HI	Control Group NH
Total population	*n* = 58 (100%)	*n* = 173 (100%)
Gender		
Boy	33 (57%)	89 (51%)
Girl	25 (43%)	84 (49%)
Technology		
CI	39 (67%)	
HA	16 (28%)	
Bahs	3 (5%)	
Diagnosis of HI		
Connexin 26	6 (10%)	
Pendred’s syndrome	5 (9%)	
CMV	5 (9%)	
Other syndromes	5 (9%)	
Meningitis	2 (3%)	
Anotia/Microtia	1 (2%)	
Auditory Neuropathy	1 (2%)	
Other	3 (5%)	
Degeneratio labyrinthi acustici non specificata	30 (52%)	
Median age at diagnosis		
CI (months)	6	
HA/Bahs (months)	7.5	
Additional disabilities		
Visual disabilities	1 (2%)	
Speech output disabilities	1 (2%)	
Attention Deficit/hyperactivity disorder (ADHD)	1 (2%)	
Medical disabilities	7 (12%)	
Autism spectrum disorder	3 (5%)	
Cerebral palsy	1 (2%)	
Developmental delay	2 (4%)	
CHARGE syndrome	1 (2%)	
Noonan syndrome	1 (2%)	
Microcephaly	1 (2%)	
Asphyxia	1 (2%)	
High level of social well-being		
Year 3	36 (68%)	
Year 6	30 (68%)	
Age at start with hearing technology/device		
Median age at implantation with CI (months)	13	
Median age at start with HA/Bahs (months)	13	

**Table 2:** Categories of standard scores vs. level of social well-being and device at year 3 and 6.

		Standard Score PPVT-4			Chi-Square *p*-Value
		<85	85–100	>100	
Social well-being year 3	Low	1 (8%)	3 (23%)	9 (69%)	0.1602
	High	3 (9%)	4 (12%)	27 (79%)	
Social well-being year 6	Low	0	8 (62%)	5 (38%)	0.715
	High	0	10 (36%)	18 (64%)	
Device year 3	CI	3 (10%)	5 (16%)	23 (74%)	0.245
	HA/Bahs	1 (6%)	2 (13%)	13 (81%)	
Device year 6	CI	0	10 (36%)	18 (64%)	0.447
	HA/Bahs	0	7 (50%)	7 (50%)	

**Table 3:** Children with and without additional disabilities and level of social well-being at year 3 and 6.

		No Additional Disabilities	Additional Disabilities	Chi-Square *p*-Value
Social well-being year 3	Low	8 (24%)	9 (47%)	0.0746
	High	26 (76%)	10 (53%)	
Social well-being year 6	Low	5 (17%)	9 (60%)	0.0039 *
	High	24 (83%)	6 (40%)	

* *p* < 0.05.

## Data Availability

The data presented in this study are available on request from the corresponding author. The data are not publicly available due to general data protection rules (GDPR).
